# Altered Brain Network Connectivity as a Potential Endophenotype of Schizophrenia

**DOI:** 10.1038/s41598-017-05774-3

**Published:** 2017-07-14

**Authors:** Peng Li, Teng-Teng Fan, Rong-Jiang Zhao, Ying Han, Le Shi, Hong-Qiang Sun, Si-Jing Chen, Jie Shi, Xiao Lin, Lin Lu

**Affiliations:** 1 Peking University Sixth Hospital, Peking University Institute of Mental Health, Key Laboratory of Mental Health, Ministry of Health (Peking University), National Clinical Research Center for Mental Disorders (Peking University Sixth Hospital), Beijing, 100191 China; 2 Peking-Tsinghua Center for Life Sciences and PKU-IDG/McGovern Institute for Brain Research, Peking University, Beijing, 100871 China; 3 Department of Alcohol and Drug Dependence, Beijing Hui-Long-Guan Hospital, Peking University, Beijing, 100096 China; 4 National Institute on Drug Dependence and Beijing Key laboratory of Drug Dependence, Peking University, Beijing, 100191 China

## Abstract

Abnormal functional brain connectivity could be considered an endophenotype of psychosis in schizophrenia. Identifying candidate endophenotypes may serve as a tool for elucidating its biological and neural mechanisms. The present study investigated the similarities and differences of features of brain network connectivity between patients and their first-degree relatives. Independent component analysis was conducted on imaging data collected from 34 healthy controls, 33 schizophrenia patients, and 30 unaffected first-degree relatives. The correlation between functional connectivity with neurocognitive performance and clinical symptoms were calculated. Abnormalities of between-network connectivity largely overlapped in patients and first-degree relatives, but the extent of such abnormalities was relatively minor in relatives. Negative connectivity between language networks and executive control networks was impaired in schizophrenia patients and their first-degree relatives, and this decreased connectivity was correlated with performance in language processing. Similar impairments were found in high-visual network and executive network coupling, and this decreased connection was correlated with the severity of positive symptoms in patients. The results indicated that abnormal functional connectivity within and between perceptual systems (i.e., high-visual and language) and executive control networks was related to the generic risk of schizophrenia, which makes it a potential endophenotype for schizophrenia.

## Introduction

Schizophrenia is one of the most complex and heterogeneous mental disorders. It causes impairments in multiple cognitive domains, including perception, attention, and executive function. Traditional genetic research that uses linkage approaches has identified multiple susceptibility alleles in schizophrenia^1, 2^. It has been suggested that the genetic risk for clinical disorders must be mediated by abnormalities of biological traits or endophenotypes^3, 4^. Another method to investigate genetic risk in psychotic disorders is based on identifying endophenotypes. Endophenotypes are intermediate phenotypes that can be detected in both psychotic patients and their first-degree relatives and can parse the heterogeneity of psychotic disorders to provide a more reliable diagnostic index than the illness itself^5, 6^. The concept of endophenotypes was first proposed by Gottesman and Shields, the essential traits of which are heritability and illness state independence. For the development of therapeutics, biomarkers or endophenotypes must be identified and used to refine the diagnostic system.

Previous studies characterized schizophrenia as a functional disconnection syndrome, in which disruptions of connectivity are associated with structural and functional aspects and impairment of the integrity of white matter (WM) fiber tracts^7–11^. Some researchers have proposed that impairments in resting brain functional connectivity can be used as an endophenotype of schizophrenia^12, 13^. The essential feature of an endophenotype is heritability. Previous twin studies have demonstrated that magnetic resonance imaging (MRI) measures of brain functional network organization are indeed heritable^14^. Furthermore, the heritable feature of an endophenotype also implies that biomarkers that are found in schizophrenia patients should also be found in their first-degree relatives^4^. However, most imaging studies have focused solely on schizophrenia patients, with relatively little emphasis on abnormalities of connectivity in first-degree relatives.

First-degree relatives of schizophrenia patients do not present the illness but share an average of 50% of the genes of schizophrenia patients, including schizophrenia-risk genes^4, 15^. Such first-degree relatives also have a 10-fold higher risk of developing schizophrenia^16^. Studies of unaffected relatives can provide additional information because of the instability and medical condition of patients with schizophrenia. A few meta-analyses have been conducted to determine the magnitude and extent of brain volume differences in first-degree relatives of schizophrenia patients^17, 18^. Neuroimaging studies of unaffected relatives of schizophrenia patients have revealed basal ganglia network abnormalities that are shared by both patients and their relatives^17–19^. A previous twin study that utilized positron emission tomography found an increase in caudate dopamine D_2_ receptor availability in unaffected co-twins of patients with schizophrenia^20^. Additionally, functional connections between fronto-premotor and meso/paralimbic networks were also reduced in both schizophrenia patients and their relatives^21^. Alterations in functional connectivity reflect alterations in synaptic efficacy and may characterize schizophrenia diathesis. However, most studies have used seed analysis, which uses a predefined voxel or region of interest (ROI). The results of these researches may thus be biased by selecting specific brain ROIs^11^.

An emerging analysis technique involves independent component analysis (ICA), which is entirely data-driven. Every independent component is an independent source signal that represents a unique spatial pattern of changes in blood oxygen level-dependent (BOLD) signals and coherent groupings of functional MRI (fMRI) activation^22^. Every component has a time-course that is temporally coherent, and the involved brain areas that share similar time-courses are defined as an ICA component^21^. In addition to deficient connectivity within different functional components, interactions between networks are also very important but can be easily neglected. Such interactions may be quantified to reflect how one specific functional network is influenced by another and can provide more information to test the hypothesis of dysconnectivity in schizophrenia^23^. In the present study, we calculated interrelationships across different functional brain networks. If functional connectivity abnormalities is proven to be a schizophrenia endophenotype, then it may serve as a measureable genetic risk factor. Future studies, therefore, could search for the expression of genes that are associated with brain network disorganization rather than schizophrenia genes *per se*. Studying relatives of probands is necessary to verify candidate endophenotypes of psychosis.

The perception of the world is distorted in patients with schizophrenia. Hallucinations are the most persistent and treatment-resistant symptom of schizophrenia^24^. However, most research has focused on self-monitoring- and self-regulation-related brain areas, and few studies have focused on impairments in sensory processing systems. The present study focused on the somatosensory system, the cognitive control system, and the interactions between these networks. Language processing has also been shown to be impaired in schizophrenia. Therefore, the present study also employed a verbal fluency task. The first hypothesis of the present study was that there is dysconnectivity within and between perception neural networks and executive control networks in patients with schizophrenia, and the dysconnectivity is related to positive symptoms. The second hypothesis was that unaffected first-degree relatives share some overlapping abnormalities with schizophrenia patients, thus making dysconnectivity a candidate endophenotype of schizophrenia.

## Results

### Demographics

Demographic and clinical data are summarized in Table 1. No significant differences in age or gender were found among groups (age: *F*_(2,94)_ = 2.63, *p* = 0.08; sex: *χ²* = 0.59, df = 2, *p* = 0.745). The cognitive test results are presented in Table 1. Compared with healthy controls, patients with schizophrenia had significantly lower scores on the verbal fluency test (*p* < 0.05). Verbal fluency scores did not differ significantly between first-degree relatives and healthy controls.

**Table 1:** Demographic and clinical characteristics of the participants in each group.

Characteristic	SZ	HC	FDR	*p*
	*n* = 33	*n* = 34	*n* = 30	
	Mean (SD)	Mean (SD)	Mean (SD)	
Age (years)	30.60 (8.13)	28.12 (6.5)	32.4 (7.87)	0.08
Sex (male/female)	11/22	14/20	10/20	0.75
Education (years)	12.36 (2.68)	12.74 (3.79)	11.67 (3.95)	0.53
Age at onset (years)	26.21 (8.242)	NA	NA	
Length of illness (years)	4.74 (2.52)	NA	NA	
PANSS score				
Total	78.36 (7.95)	NA	NA	
Positive	25.61 (3.41)	NA	NA	
Negative	17.15 (2.81)	NA	NA	
General	35.60 (4.15)	NA	NA	
Verbal fluency				
Total	18.03 (6.47)	22.97 (5.14)	21.23 (5.14)	0.00
Correct	17.3 (5.98)	19.93 (5.17)	21.65 (5.13)	0.00

SZ, schizophrenia patients; FDR, first-degree relatives; HC, healthy controls.

### Network function

**Figure 1:**
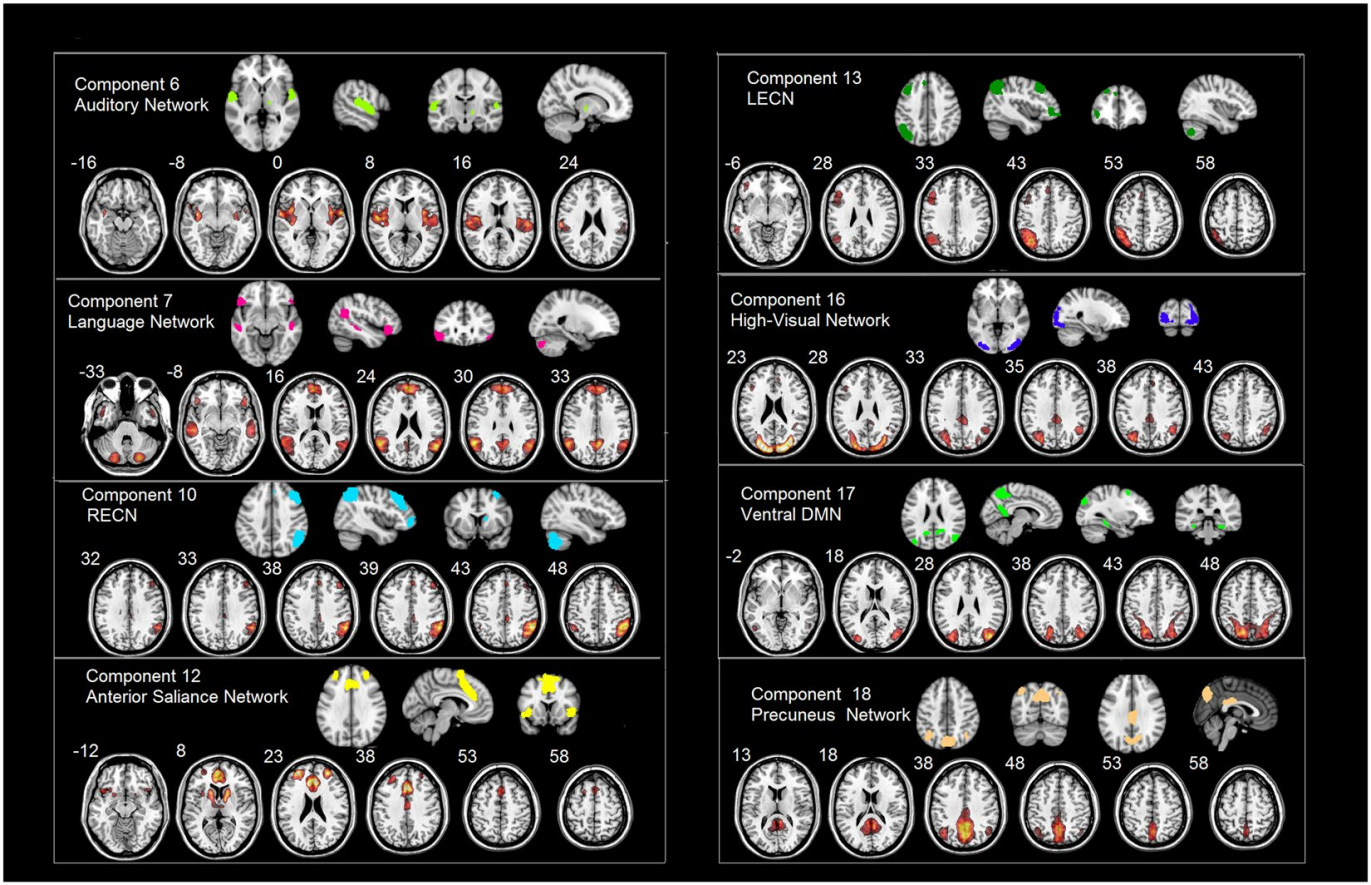
Non-artifactual network templates and corresponding components identified across all of the participants. Component 6 and auditory network template: *R* = 0.37. Component 7 and language network template: *R* = 0.48. Component 10 and RECN template: *R* = 0.54. Component 12 and anterior salience network template: *R* = 0.42. Component 13 and LECN template: *R* = 0.56. Component 16 and HVN template: *R* = 0.45. Component 17 and ventral DMN template: *R* = 0.37. Component 18 and precuneus template: *R* = 0.59. SZ, schizophrenia patients; FDR, first-degree relatives; HC, healthy controls; RECN, right executive control network; LECN, left executive control network; DMN: default mode network.

The ICA identified six functional brain networks of interest: (*i*) auditory network, (*ii*) language network, (*iii*) high-visual network, (*iv*) executive control network, (*v*) anterior salience network, and (*vi*) ventral default mode network (vDMN). The reliability of these brain network templates have been carefully evaluated by many research groups (http://findlab.stanford.edu/functional_ROIs.html), and the components that were selected to represent the specific functional networks had the highest correlation with the prior templates (Fig. 1).

### Within-network connectivity

**Figure 2:**
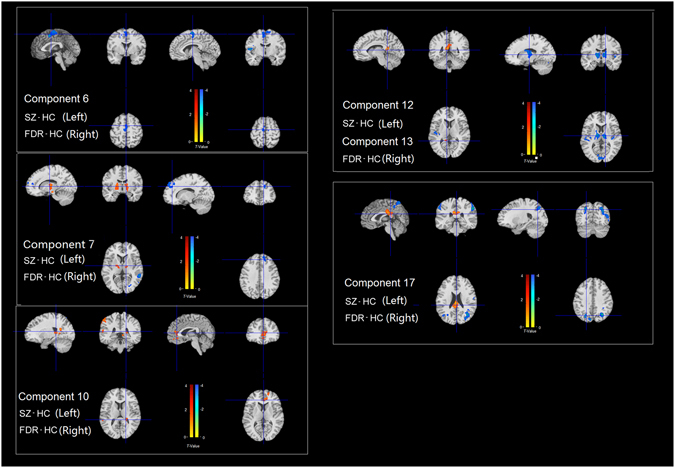
Brain areas that showed differences in separate brain networks in patients with schizophrenia and their first-degree relatives compared with healthy controls. The precise coordinates are presented in Supplementary Table S1. SZ-HC: differences in functional connectivity within the network between patients and healthy controls. FDR-HC: differences in functional connectivity within the network between first-degree relatives and healthy controls. The statistical threshold was *p* < 0.05, alphasim corrected. SZ, schizophrenia patients; FDR, first-degree relatives; HC, healthy controls.

The one-way ANOVA of within-network connectivity included the schizophrenia patient group, first-degree relative group, and healthy control group as between-subject factors and network as the within-subject factor. ANOVAs were conducted separately for different networks, followed by *post hoc* tests. We then used alphasim correction (*p* < 0.05) for multiple comparisons within each network. The results are shown in Fig. 2 and Supplementary Table S1.

#### Auditory network

Compared with healthy control subjects, schizophrenia patients exhibited a decrease in connectivity in the cingulate gyrus within the auditory network. In unaffected first-degree relatives, a decrease in connectivity was found in the bilateral insula and middle frontal gyrus.

#### Language network

Compared with the healthy control group, schizophrenia patients exhibited an increase in connectivity in the bilateral thalamus and a decrease in connectivity in the fusiform gyrus, bilateral frontal gyrus, and bilateral temporal gyrus. First-degree relatives also exhibited some impairment compared with healthy controls, but to a slightly lesser degree compared with schizophrenia patients. In first-degree relatives, impairments were found in the right superior frontal gyrus and right medial temporal gyrus. No increase in connectivity was found in first-degree relatives.

#### Executive control network

Consist of right executive control network (RECN) and left executive control network (LECN). In addition to an increase in connectivity in the inferior parietal lobule and parahippocampal gyrus, a decrease in connectivity was found in the bilateral insula and precuneus in schizophrenia patients compared with healthy controls. First-degree relatives presented a similar increase in connectivity in the inferior parietal lobule but no decreases in connectivity.

#### Anterior salience network

Patients with schizophrenia presented a decrease in connectivity in the bilateral insula. No abnormalities was found in first-degree relatives.

#### Ventral default mode network (DMN)

Schizophrenia patients and first-degree relatives presented some overlap in deficits in the DMN. Connectivity in the precuneus and inferior partial lobule decreased in both groups. Schizophrenia patients also presented a decrease in connectivity in the inferior parietal lobule and superior occipital gyrus and an increase in connectivity in the post cingulate and inferior parietal lobule.

Similar patterns of changes within several functional networks were found in patients and first-degree relatives, especially in the language network, executive network, and DMN, although to a slightly lesser extent in first-degree relatives (Fig. 2, Supplementary Table S1).

### Between-network connectivity

**Figure 3:**
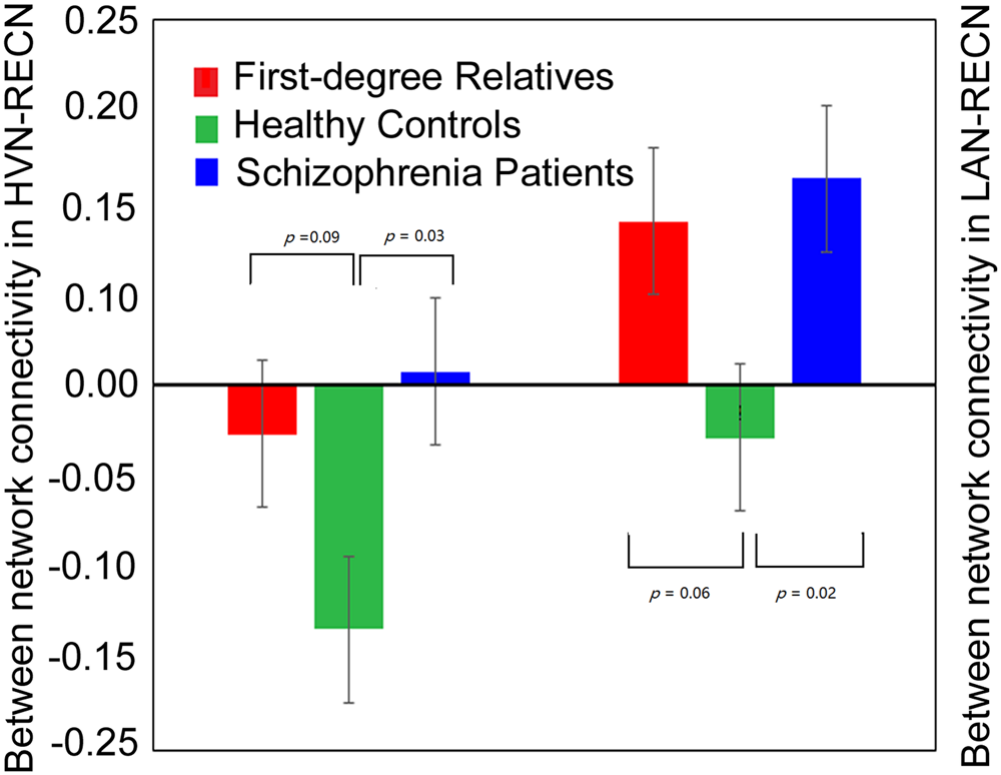
Bar plots for significant differences in intermodule connectivity between perception networks (high-visual and language) and right executive control network. RECN, right executive control network; LAN, language network; AUDN, auditory network; HVN, high-visual network; SZ, schizophrenia patients; FDR, first-degree relatives; HC, healthy controls. *p* value represent the uncorrected significant level of the comparison.

We analyzed interactions between different networks. Between-network connectivity was calculated using average time-courses from different functional networks, especially networks that are related to the positive symptoms of schizophrenia. Previous studies showed that the deficits of right-brain regions in executive control network are more dominant in schizophrenia patients^25^ and in the unaffected offspring^26^, thus our interests are focused on the connectivity of right executive control network. When examining differences in between-network connectivity across the three groups, we observed a significant decrease in connectivity between perception networks (i.e., high-visual and language) and cognitive control network (RECN) in schizophrenia patients compared with healthy controls and a trend toward a decrease in connectivity in first-degree relatives compared with healthy controls (Fig. 3).

**Figure 4:**
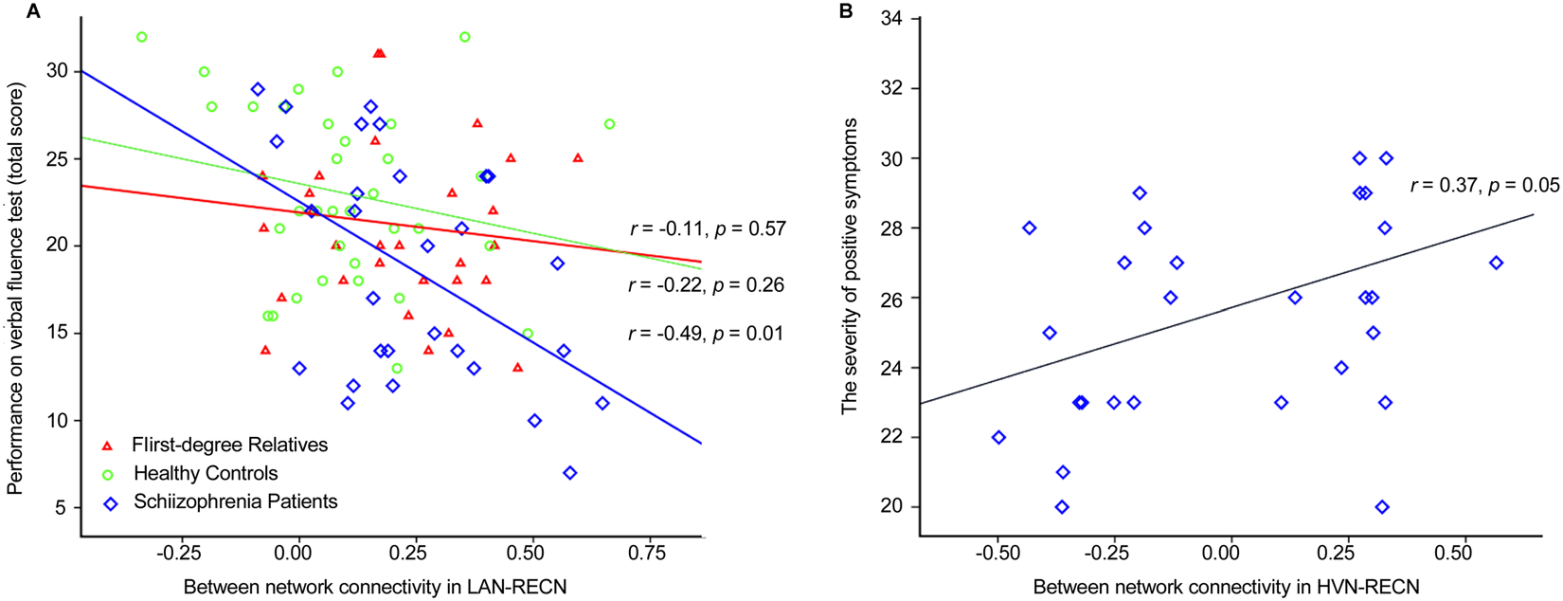
Relationship between internetwork connectivity measures and clinical and cognitive variables. (**A**) Correlation between connectivity between the language and executive control networks and language processing performance. (**B**) Correlation between connectivity between the high-visual network and executive control networks and severity of positive symptoms. RECN, right executive control network; LAN, language network; HVN, high-visual network.

To verify that disruptions of between-network connectivity are involved in schizophrenia, we analyzed correlations between impairments in coupling and the severity of symptoms, especially positive symptoms, and cognitive performance on the verbal fluency task. In patients with schizophrenia, a negative correlation was found between RECN/language network coupling and language processing (*r* = −0.49, *p* = 0.01; Fig. 4A). The greater disruption of top-down regulation resulted in worse performance on the language processing task. However, this correlation was not found in first-degree relatives. Functional connectivity between the high-visual network and RECN was positively correlated with the severity of positive symptoms (*r* = 0.37, *p* = 0.05; Fig. 4B).

## Discussion

The present study identified overlapping and unique functional connectivity abnormalities in patients with schizophrenia and their unaffected first-degree relatives. We provided evidence that supports the theory that dysconnectivity might be a candidate endophenotype of schizophrenia. Using a combination of ICA and analyses of functional network connectivity correlations, we extracted six independent brain networks, all of which resembled networks that were previously identified in other resting-state studies that performed ICA^24^. The present study focused on the interaction between executive networks and sensory-related functional networks, which could provide further insights into the generation of hallucination.

Consistent with previous studies, we identified several abnormal functional network connections in schizophrenia patients. The DMN has been shown to be involved in monitoring processes, such as attention to internal emotional states^27^ and self-referential processing^28^. Many studies revealed that impairments in connectivity in the DMN are related to difficulties in disengaging attention from internal states and refocusing on external information^27^. However, a mixed pattern of increases and decreases in connectivity within the DMN has been reported in patients with schizophrenia^29, 30^. The present study also identified some abnormalities in the DMN in patients with schizophrenia. The posterior cingulate and inferior parietal lobule presented hyperconnectivity in the ventral DMN in patients with schizophrenia and no enhancement of connectivity in first-degree relatives. Additionally, a decrease in connectivity between the precuneus system and ventral DMN was detected in patients with schizophrenia.

An essential feature of an endophenotype is that it is heritable, meaning that it can be detected in both patients and first-degree relatives, even when first-degree relatives do not present symptoms of the illness. We found that patients with schizophrenia and their first-degree relatives presented impairments in connectivity within specific networks and also impairments in connectivity between networks. We found that schizophrenia patients and their first-degree relatives had similar dysconnectivity in the RECN and vDMN, and this dysfunction was related to the symptoms of schizophrenia. The RECN, vDMN, and language network are thought to be key networks that are involved in cognitive control and perception processing. A previous fMRI study suggested that the core mechanism that produces hallucinations involves not a single pathway but rather a more complex functional loop that comprises auditory and language networks and the left inferior frontal gyrus^31^. Several studies have examined the relationship between abnormal functional connectivity and the positive symptoms of schizophrenia. One hypothesis is that the positive symptoms are instances of inner speech that is misidentified as deriving from the outer environment because of disruptions of projections that signal to sensory systems that actions (and thought) are self-generated^32, 33^. Numerous neuroimaging studies have suggested that hallucinations are associated with structural and functional abnormalities in a widely distributed set of brain networks, such as the lateral prefrontal cortices, the cingulate cortex, and regions of the temporal lobes. These regions are involved in language, attention, executive function, and memory^34^. Patient ratings of experiences of hallucinations were found to be positively correlated with functional coupling that links the left inferior frontal gyrus (IFG) with the bilateral auditory cortex, right posterior temporal lobe, middle right anterior cingulate cortex, right ventral striatum, and left nucleus accumbens^35^.

Auditory hallucinations have been reported to be related to the aberrant modulation of the auditory cortex by anterior midline DMN regions and excessive functional coordination between the putamen and Wernicke’s area^33^. In the present study, hyperconnectivity between the bilateral thalamus and language network could be the means by which the lower threshold-to-consciousness of conversational language representations occurs in patients with schizophrenia. The thalamus is located in the forebrain, which is superior to the midbrain and near the center of the brain. The thalamus projects nerve fibers to the cerebral cortex in all directions^36^. A major role of the thalamus is to support motor and language systems. The connectivity of the thalamus in the language system was shown to be enhanced in patients with schizophrenia. The thalamus also plays a major role in regulating the level of consciousness. Thalamic nuclei have strong reciprocal connections with the cerebral cortex, thus forming thalamo-cortico-thalamic circuits that are believed to be involved in consciousness^37^. The decrease in connectivity of the cingulate gyrus in the auditory network and decrease in connectivity of the frontal gyrus in the language network imply a functional breakdown of appropriate monitoring of sensory information processing. In addition to dysconnectivity within perception networks, our functional network connectivity analysis found that the negative correlation between the language network and executive network was reversed in patients with schizophrenia and their first-degree relatives. This finding implies that top-down regulation was disrupted, and this disruption was positively correlated with language processing performance in patients with schizophrenia. No such correlation was found in first-degree relatives and healthy controls. The lack of a correlation between abnormal connectivity and cognitive performance in first-degree relatives indirectly suggests that dysconnectivity may serve as an endophenotype because it is able to parse the diversity of superficial characteristics.

In addition to auditory hallucinations in schizophrenia, visual hallucinations are also distressful. In the present study, negative connectivity between the high-visual network and executive control network decreased, indicating a deficit in cognitive control from higher-level brain regions. The negative correlation between disruptions of connectivity and the severity of positive symptoms supports this supposition. The positive symptoms of schizophrenia comprise delusions and visual and auditory hallucinations, which have been shown to be related to deficits in cognitive control and the monitoring of consciousness. The aberrant monitoring or control of the high-visual network thus makes it difficult for schizophrenia patients to detect the origins of a visual stimulus, which may be a neural mechanism of visual hallucinations. Previous studies showed that both high-risk subjects and patients exhibited significant reductions of activation correlations between regions that are related to visual language processing^38^. Prefrontal-temporal disconnections were also found in schizophrenia patients^39^. In the present study, the disrupted communication between high-level executive network and the sensory related network were also found in the unaffected first-degree relatives. The abnormal dysconnectivity between perception networks and executive control networks overlapped in patients with schizophrenia and their first-degree relatives, which may support the theory that such dysconnectivity can be considered an endophenotype of schizophrenia.

The results of the present study have to be apprehended under some limitations. Firstly, the inter-network connectivity differences between FDR and HC could not survive the multiple comparison correction, and the inter-network differences between SZ and HC are only marginal significant after correction, which may undermine the potential of the brain changes to be endophenotype. Even so, the similar change patterns suggested that the dys-communication between networks might represent a candidate intermediate phenotype, and its reliability need further verification. Secondly, further twin studies are required to rule out the possibility that such network abnormalities result from shared environmental effects.

Altogether, the present study supports earlier reports of impairments in inner- and inter- network coupling in schizophrenia. These impairments were also found in first-degree relatives of schizophrenia patients. The dysconnectivity within and between essential functional networks, such as the RECN, vDMN, and language network, could be an endophenotype of schizophrenia. Studying brain network abnormalities may parse the heterogeneity of disease symptoms and provide information on genetic factors.

## Materials and Methods

### Participants

We assessed a total 97 subjects: 34 healthy controls, 33 schizophrenia patients, and 30 unaffected first-degree relatives. The groups were matched for age and sex. Diagnoses were based on detailed medical and psychiatric histories, chart reviews, and the Structured Clinical Interview for DSM Disorders. The exclusion criteria were the following: (*i*) age < 18 or >45 years, (*ii*) left handedness, (*iii*) history of brain trauma with loss of consciousness, neurological disease, or serious physical disease (e.g., respiratory disorders, cardiovascular disease, and so on), (*iv*) diagnosis of alcohol/substance abuse within 12 months before participation in the study, and (*v*) contraindications for MRI. The Ethics Committee of Beijing Hui-Long-Guan Hospital (Beijing, China) approved the study, and all of the participants provided written informed consent. The methods were carried out in accordance with the approved guidelines.

### Data acquisition and preprocessing

fMRI data were acquired using a 3.0 Tesla Magnetom Trio scanner. The resting-state functional scans were obtained using a gradient-recalled echo-planar imaging sequence that was sensitive to BOLD contrast (repetition time, 2000 ms; echo time, 30 ms; flip angle, 90°). The slice thickness was 4 mm (no gap) with a matrix size of 64 × 64 and field of view of 220 × 220 mm², resulting in a voxel size of 3.4 × 3.4 × 4.0 mm³. Each brain volume comprised 33 axial slices, and each functional run contained 240 image volumes. During data acquisition, the subjects were instructed to close their eyes, relax, and remain awake. All of the images were checked for artifacts, structural abnormalities, and pathologies by a qualified neuroradiologist.

Image preprocessing was performed using statistical parametric mapping (SPM8) software (http://www.fil.ion.ucl.ac.uk/spm/software/spm8/). To allow for magnetization equilibrium, the first 20 volumes of the functional images were discarded. The preprocessing procedure included slice-timing correction and head-motion correction. Four patients with schizophrenia were excluded because of head motion (>2.5 mm). Each fMRI scan was intensity-scaled to yield a whole-brain mean value of 10000. Temporal band-pass filtering (0.01 < f < 0.08 Hz) was then performed, and the time series in WM and cerebrospinal fluid (CSF) and six affine motion parameters were regressed from the data. The removal of linear and quadratic trends was also implemented. To obtain results at the group level, single-subject images were nonlinearly normalized to Montreal Neurological Institute (MNI) space using DARTEL in SPM8 and resampled to 3 × 3 × 3 mm³ cubic voxels. Finally, the data were spatially smoothed with a 6 mm full width at half-maximum (FWHM) Gaussian kernel.

### Group independent component analysis and component selection

Group ICA Toolbox 3.0a software (http://mialab.mrn.org/software/gift/) was used to decompose imaging data into functional networks. The initial data reduction was performed using principal component analysis (PCA), followed by an independent component (IC) estimation that produced time-courses and spatial maps using the FastICA algorithm. Twenty ICs were estimated because this number has been shown to efficiently decentralize data^40^. Each component’s time-course represented a pattern of synchronized brain activity whose coherency patterns across voxels were represented in the associated spatial map.

To select components, a standard method was used to reject artifactual networks. Spatial maps of each component were correlated with prior probabilistic maps of gray matter (GM), WM, and CSF within a standardized brain space that is provided by MNI templates in SPM8 (spm8/tpm/csf.nii, grey.nii, and white.nii)^41^. Eleven components that showed relatively high correlations with WM and CSF and low correlations with GM were considered artifacts and discarded. The thresholds for spatial correlations for the CSF, WM, and GM were set at *r²* < 0.025, *r²* < 0.02, and *r²* > 0.025, respectively. Components 5 and 8 were associated with head motion. Components 9 and 20 were associated with the cerebral ventricle (see Supplementary Fig. S1). Statistical parametric maps using one-sample *t*-tests were created for each remaining component to further examine validity, and the remaining components underwent correlation analysis with prior template networks, which have been well addressed in numerous studies (htttp://findlab.standord.edu/functional_ROIs.html). The selection of corresponding networks depended on the correlation coefficients. Components with a higher correlation with the prior template were included as a function network. Eight identified networks presented relatively high correlations with the templates.

### Group comparisons of functional connectivity within independent component analysis components

The remaining components were subjected to one-way analysis of variance (ANOVA), with controls, schizophrenia patients, and their unaffected first-degree relatives as three independent factors, to identify significantly anomalous patterns of functional connectivity. *Post hoc* tests and AlphaSim correction were conducted to examine group differences in functional networks, with a statistical threshold of *p* < 0.05. The high-dynamic range images that were generated by these processes were viewed in the NeuroElf 09c toolbox (http://www.neuroelf.net/).

### Group comparisons of functional network connectivity across independent component analysis components

Time-course data were band-pass-filtered using a Butterworth filter with cut-off frequencies of 0.008-0.15 Hz, and each network’s time-courses were subjected to functional network connectivity analysis using the Functional Connectivity network toolbox (http://mialab.mrn.org/software/fnc/documentation.html). The focus of the present study was on identifying interrelationships between perception networks (i.e., language network, high-visual network, and auditory network) and executive control networks (RECN & LECN). Two-sample *t*-tests were conducted separately to identify changes in between-network connectivity in schizophrenia patients and first-degree relatives compared with healthy controls. Correlations between interconnectivity values and clinical symptom severity and verbal fluency performance were analyzed followed.

## Electronic supplementary material

supplementary informaiton
