# Supplementary material for: Altered Brain Network Connectivity as a Potential Endophenotype of Schizophrenia
Source: Sci Rep. 2017 Jul 14;7:5483. doi: 10.1038/s41598-017-05774-3 (PMC5511161; doi:10.1038/s41598-017-05774-3)

**Altered Brain Network Connectivity as a Potential Endophenotype of Schizophrenia**

**Peng Li, M.D., Ph.D.,^1, #^ Teng-Teng Fan, M.D., ^1, #^ Rong-Jiang Zhao, M.D.,^3^ Ying Han, Ph.D.,^4^ Le Shi, M.D., ^1, 4^ Si-Jing Chen, M.D.,^1^ Hong-Qiang Sun, M.D.,^1^ Jie Shi, M.D., Ph.D., ^4^ Xiao Lin,^1, 2^ * Lin Lu, M.D., Ph.D.^1, 2, 4,^ ***

^1^Peking University Sixth Hospital, National Clinical Research Center for Mental Disorders, Peking University Institute of Mental Health, Key Laboratory of Mental Health, Ministry of Health, Peking University, Beijing 100191, China

^2^Peking-Tsinghua Center for Life Sciences and PKU-IDG/McGovern Institute for Brain Research, Peking University, Beijing 100871, China

^3^Department of Alcohol and Drug Dependence, Beijing Hui-Long-Guan Hospital, Peking University, Beijing 100096, China

^4^National Institute on Drug Dependence and Beijing Key laboratory of Drug Dependence, Peking University, Beijing 100191, China

^#^Equal contribution

*Corresponding author:

Lin Xiao

Peking-Tsinghua Center for Life Sciences and PKU-IDG/McGovern Institute for Brain Research, Peking University

5 Yiheyuan Road, Haidian District, Beijing 100871, China

E-mail: 1501111480@pku.edu.cn

Prof. Lin Lu

Peking University Sixth Hospital/Peking University Institute of Mental Health/National Clinical Research Center for Mental Disorders, Peking University,

51 Huayuan Bei Road, Haidian District, Beijing 100191, China

E-mail: linlu@bjmu.edu.cn

Tel: +86-10-82805308

Fax: +86-10-62032624

**Supplementary Table 1.** Clusters showed alterations in within-network connectivity in the SZ, FDR, and HC groups (*post hoc* *t*-test).

| Anatomical region |  | Coordinates |  | *t* | Cluster size | Hemisphere | Brodmann area |
| --- | --- | --- | --- | --- | --- | --- | --- |
| **SZ > HC** | X | Y | Z |  |  |  |  |
| **Language network (Component 7)** |  |  |  |  |  |  |  |
| Thalamus | 16 | -14 | -6 | 2.53 | 120 | Right | 10 |
| Thalamus | -20 | -11 | -6 | 2.51 | 120 | Left | 10 |
| **Right ECN (Component 10)** |  |  |  |  |  |  |  |
| Inferior parietal lobule | -54 | -46 | 43 | 2.54 | 203 | Left | 40 |
| Parahippocampal gyrus | 15 | -45 | 3 | 2.60 | 129 | Right | 30 |
| **Left ECN (Component 13)** |  |  |  |  |  |  |  |
| Posterior cingulate | -6 | -44 | 13 | 2.65 | 118 | Left | 29 |
| **Ventral DMN (Component 17)** |  |  |  |  |  |  |  |
| Posterior cingulate | -9 | -35 | 22 | 2.68 | 272 | Left | 23 |
| Inferior parietal lobule | 50 | -65 | 38 | 2.63 | 120 | Right | 39 |
| **FDR > HC** |  |  |  |  |  |  |  |
| **Right ECN (Component 10)** |  |  |  |  |  |  |  |
| Inferior parietal lobule | -54 | -43 | 43 | 2.69 | 135 | Left | 40 |
| Superior frontal gyrus | 22 | 55 | 5 | 2.48 | 154 | Right | 10 |
| **SZ < HC** |  |  |  |  |  |  |  |
| **Auditory network (Component 6)** |  |  |  |  |  |  |  |
| Cingulate gyrus | -4 | 3 | 44 | -2.53 | 251 | Left | 24 |
| **Language network (Component 7)** |  |  |  |  |  |  |  |
| Fusiform gyrus | 34 | -67 | -7 | -2.67 | 140 | Right | 19 |
| Medial frontal gyrus | -8 | 44 | 27 | -2.56 | 142 | Left | 9 |
| Superior frontal gyrus | 29 | 54 | 24 | -2.54 | 149 | Right | 10 |
| Superior temporal gyrus | -50 | -55 | 25 | -2.59 | 147 | Left | 39 |
| Middle temporal gyrus | -23 | -73 | -27 | -2.47 | 233 | Right | 22 |
| **ECN (Component 10)** |  |  |  |  |  |  |  |
| Precuneus | 36 | -68 | 39 | -2.58 | 221 | Right | 19 |
| Insula | 41 | 2 | 4 | -2.41 | 143 | Right | 13 |
| Insula | -40 | -35 | 19 | -2.45 | 189 | Left | 13 |
| **Anterior salience (Component 12)** |  |  |  |  |  |  |  |
| Thalamus | -15 | -12 | 12 | -2.63 | 149 | Left |  |
| Thalamus | 9 | -20 | 17 | -2.561 | 247 | Right |  |
| Superior temporal gyrus | 51 | -17 | 5 | -2.49 | 109 | Right | 22 |
| Superior temporal gyrus | -53 | -27 | 14 | -2.49 | 104 | Left | 41 |
| Inferior parietal lobule | 53 | -44 | 24 | -2.42 | 108 | Right | 40 |
| **Ventral DMN (Component 17)** |  |  |  |  |  |  |  |
| Precuneus | -6 | -62 | 52 | -2.52 | 532 | Left | 7 |
| Superior occipital gyrus | 33 | -77 | 26 | -2.65 | 552 | Right | 19 |
| Inferior parietal lobule | -46 | -39 | 51 | -2.54 | 175 | Left | 40 |
| **FDR < HC** |  |  |  |  |  |  |  |
| **Auditory network (Component 6)** |  |  |  |  |  |  |  |
| Insula | -48 | -15 | 12 | -2.45 | 211 | Left | 13 |
| Insula | 45 | -38 | 18 | -2.44 | 107 | Right | 13 |
| Medial frontal gyrus | -7 | -9 | 59 | -2.51 | 110 | Left | 6 |
| **Language network (Component 7)** |  |  |  |  |  |  |  |
| Superior frontal gyrus | 12 | 48 | 40 | -2.52 | 146 | Right | 8 |
| Superior frontal gyrus | 12 | 48 | 40 | -2.52 | 146 | Right | 8 |
| Middle temporal gyrus | 59 | -60 | 13 | -2.47 | 121 | Right | 39 |
| **Ventral DMN (Component 17)** |  |  |  |  |  |  |  |
| Precuneus | -25 | -69 | 35 | -2.55 | 156 | Left | 19 |
| Precuneus | 34 | -69 | 32 | -2.54 | 283 | R | 19 |
| Inferior Parietal Lobule | 47 | -48 | 49 | -2.57 | 104 | R | 40 |

Coordinates are given for the maximally significant voxel in each area. The statistical thresholds were set at *p* < 0.05, alphasim corrected. The coordinates are in Montreal Neurological Institute (MNI) space. The brain regions were automatically identified by NeuroElf software (<http://Neuroelf.net>). SZ, schizophrenia patients; FDR, first-degree relatives; HC, healthy controls; ECN, executive control network; DMN, default mode network.

**Supplementary Figure 1.** Four components obtained using ICA which were associated

with head motion (Comp 5 and Comp 8) and cerebral ventricle (Comp 9 and Comp 20). Threshold was *p* < 0.05, alphasim corrected.


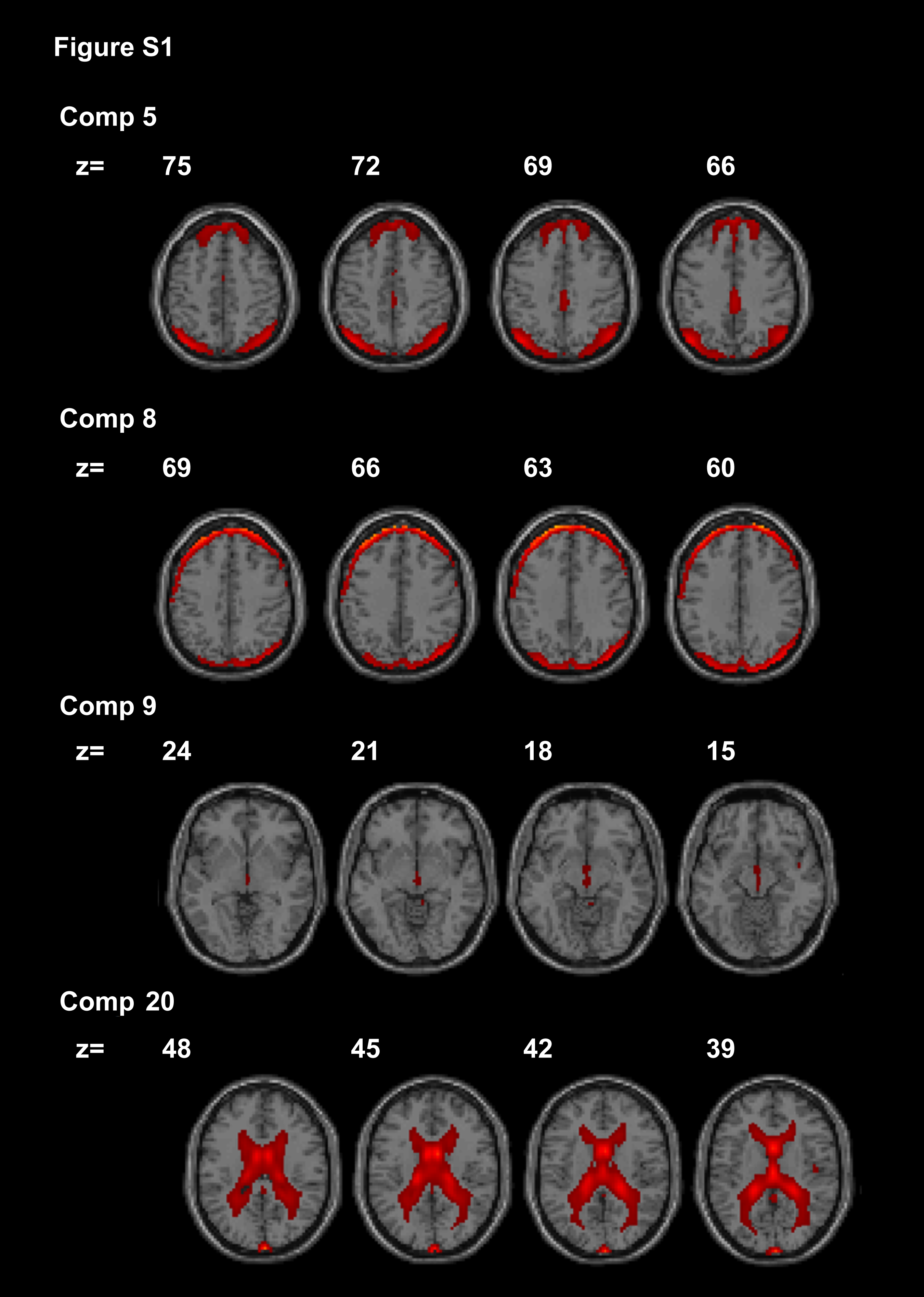

Supplement: Supplementary file 1 — supplementary informaiton [file 41598_2017_5774_MOESM1_ESM.docx]
